# Plasma β‐hydroxybutyrate concentrations in young adult females after a high‐fat meal under normoxaemia, intermittent hypoxaemia and continuous hypoxaemia: An exploratory analysis

**DOI:** 10.1113/EP093829

**Published:** 2026-07-24

**Authors:** Nicholas Goulet, Alexanne Larocque, Caroline Marcoux, Vincent Bourgon, Jean‐François Mauger, Ruwan Amaratunga, Pascal Imbeault

**Affiliations:** ^1^ Behavioural and Metabolic Research Unit, School of Human Kinetics, Faculty of Health Sciences University of Ottawa Ottawa Ontario Canada; ^2^ Laboratoire du Sommeil, Département de psychoéducation et de psychologie Université du Québec en Outaouais Gatineau Québec Canada; ^3^ Institut du Savoir Montfort Montfort Hospital Ottawa Ontario Canada

**Keywords:** high altitude, ketones, obstructive sleep apnoea

## Abstract

Hypoxaemia occurs in intermittent forms, such as obstructive sleep apnoea, and in continuous forms, such as at high altitude, and is increasingly recognized as a modulator of cardiometabolic risk. Although hypoxaemia alters postprandial glucose and lipid metabolism, its effects on ketone bodies remain unclear. Using a randomized crossover design, we examined whether 6 h of normoxaemia or intermittent hypoxaemia (15 hypoxaemic cycles/h targeting ∼85% peripheral oxyhaemoglobin saturation with 100% medical‐grade nitrogen) alters plasma β‐hydroxybutyrate (BHB) concentrations in 12 young adult females (mean [SD]: 21 [3] years) following a high‐fat meal (33% of estimated daily energy requirements; 59% of energy from fat). In a follow‐up session, a subset (*n* = 8) completed 6 h of continuous hypoxaemia (fraction of inspired oxygen ∼12.0% in a normobaric chamber). Postprandial data were analysed using baseline‐adjusted linear mixed‐effects models, with Bonferroni post hoc tests. A time × condition interaction (*P* = 0.010) indicated that BHB concentrations at 360 min were higher during continuous hypoxaemia (0.247 mmol/L; 95% CI: 0.218–0.275) than normoxaemia (0.176 mmol/L; 95% CI: 0.153–0.200; *P*_Bonferroni_ = 0.029) and intermittent hypoxaemia (0.163 mmol/L; 95% CI: 0.139–0.186; *P*_Bonferroni_ = 0.002), representing increases of 13.0% and 14.2% in estimated marginal means, respectively. This response was accompanied by higher postprandial plasma glucose and triglyceride concentrations during continuous hypoxaemia than during normoxaemia and intermittent hypoxaemia (*P*_Bonferroni_ ≤ 0.002), despite similar plasma insulin and non‐esterified fatty acid responses across conditions (*P* ≥ 0.081). These findings indicate that continuous hypoxaemia increases late postprandial plasma BHB concentrations in young adult females.

## Abstract (highlights)

- **What is the central question of this study?**
  What are the effects of normoxaemia, intermittent hypoxaemia and continuous hypoxaemia on plasma β‐hydroxybutyrate (BHB) concentrations in young adult females after a high‐fat meal?
- **What is the main finding and its importance?**
  Compared to normoxaemia, young adult females showed higher postprandial plasma BHB concentrations during continuous hypoxaemia, but not during intermittent hypoxaemia, despite similar changes in plasma concentrations of two main regulators of BHB production (non‐esterified fatty acids and insulin) across experimental conditions. These findings suggest that continuous hypoxaemia modifies postprandial BHB concentrations through mechanisms not fully explained by circulating non‐esterified fatty acids or insulin concentrations alone.

## 1 INTRODUCTION

Hypoxaemic conditions, such as obstructive sleep apnoea and high altitude, are increasingly recognized as modifiers of cardiometabolic risk, in part through their effects on postprandial metabolism (Chopra et al., 2017; Woolcott et al., 2015). Recurrent or sustained reductions in arterial oxygen saturation may alter substrate utilization, insulin sensitivity and lipid metabolism, processes that are particularly relevant during the postprandial period (Morin et al., 2021). While hypoxaemia‐related alterations in postprandial glucose and triglyceride metabolism are being increasingly described, their effects on postprandial ketone body dynamics in humans remain relatively unexplored. This represents an important knowledge gap, as ketone bodies may partly serve as signalling molecules that modulate cardiometabolic risk under oxidative or hypoxic stress (Stalmans et al., 2024).

β‐Hydroxybutyrate (BHB) is the predominant circulating ketone body and provides an index of hepatic lipid‐derived substrate handling (Newman & Verdin, 2017). Because BHB reflects the balance between hepatic fatty acid delivery, insulin‐mediated suppression of ketogenesis and mitochondrial oxidative capacity, it may capture aspects of metabolic regulation that are not apparent from glucose or triglyceride responses alone (Cotter et al., 2013; Puchalska & Crawford, 2017; Veech, 2004). Laboratory‐based experimental models of continuous hypoxaemia (e.g., simulated high altitude) and intermittent hypoxaemia (e.g., simulated obstructive sleep apnoea) have demonstrated measurable effects on postprandial glucose and lipid metabolism (Goulet, Marcoux et al., 2024, 2024; Morin et al., 2021). However, studies examining postprandial BHB responses to hypoxaemia are sparse, especially in humans, and direct comparisons between intermittent and continuous hypoxaemic exposures are lacking.

Our group previously demonstrated that, compared to normoxaemia, continuous hypoxaemia increased fasting plasma BHB concentrations over 6 h; however, it did not elevate BHB concentrations during a constant feeding period (Marcoux et al., 2022). Additionally, BHB concentrations remained statistically similar after 6 h of intermittent hypoxaemia under a postprandial state, compared with normoxaemia (Marcoux et al., 2022). Notably, these findings were limited to young adult males and did not directly compare intermittent and continuous hypoxaemic exposures under matched nutritional states. In parallel, we have demonstrated that females exhibit attenuated postprandial disturbances in glucose and lipid metabolism in response to intermittent hypoxaemia (Goulet, Marcoux et al., 2024). Building on this prior work, the present study examined plasma BHB responses, along with plasma concentrations of other metabolites (glucose, non‐esterified fatty acids [NEFA], triglycerides) and insulin, during 6 h of normoxaemia, intermittent hypoxaemia and continuous hypoxaemia in young adult females following the consumption of a high‐fat meal. We considered this an exploratory study to generate hypotheses for future research. As such, there were no formal hypotheses.

## 2 METHODS

### 2.1 Ethical approval

This single‐site, laboratory‐based, randomized crossover study was approved by the University of Ottawa Health Sciences and Science Research Ethics Board (ethics file number: H‐06‐18‐837) and conducted in accordance with the *Declaration of Helsinki*, except for prospective registration in a publicly accessible database. All participants provided written informed consent before enrolment. The present study was conducted as part of a larger randomized crossover trial examining the effects of hypoxaemia on postprandial metabolism in humans; detailed descriptions of the preliminary screening procedures and sample size considerations have been published previously (Goulet, Marcoux et al., 2024); key methodological elements relevant to the current analyses are reiterated here for completeness.

### 2.2 Participants

Participants were recruited between April 2022 and April 2023 using convenience sampling at the University of Ottawa (Ottawa, Canada; ∼65 m above sea level). Twelve young adult females met the eligibility criteria and completed the experimental protocol. Inclusion criteria were age 18–30 years and habitual sleep duration of 7–10 h per night. Exclusion criteria included a history of respiratory disease, hypertension, cardiovascular disease, diabetes, current tobacco smoking, use of lipid‐lowering medication, use of hormonal contraceptives, pregnancy, lactation, irregular menstrual cycles and lactose intolerance (which would have precluded consumption of the high‐fat meal).

### 2.3 Experimental design

Each participant completed one screening session (described in section 2.4) followed by two experimental sessions conducted under normoxaemia and intermittent hypoxaemia in a randomized crossover order (described in section 2.5). A third experimental session involving continuous hypoxaemia was subsequently completed by a subset of participants as part of a follow‐up session (described in section 2.6). Participants were instructed to obtain at least 7 h of sleep and to abstain from exercise, caffeine, alcohol and anti‐inflammatory medications for 36 h before each experimental session. A standardized dinner was provided and consumed approximately 12 h before arrival at the laboratory. The meal consisted of meat lasagna (390 kcal; 43% from carbohydrates, 33% from fat and 24% from protein; President's Choice, Brampton, Canada) or vegetarian macaroni and cheese (340 kcal; 60% from carbohydrates, 21% from fat and 19% from protein; President's Choice, Brampton, Canada), with a gluten‐free alternative (320 kcal; 61% from carbohydrates, 28% from fat and 11% from protein; Boosh, Surrey, Canada) provided to one participant with celiac disease. Experimental sessions began between 06.00 and 09.00 h (consistent within‐participant), conducted during the early follicular phase of the menstrual cycle (1–7 days following self‐reported menstruation), and were separated by at least one menstrual cycle. Female steroid sex hormones were measured to confirm that serum concentrations were similar between the experimental conditions and within the normal ranges for the early follicular phase (Stricker et al., 2006).

### 2.4 Screening session

During the screening session, participants completed medical and lifestyle questionnaires, including the Pittsburgh Sleep Quality Index. Height and body weight were measured using a stadiometer (Perspective Enterprises, Portage, MI, USA) and a beam scale (Tanita, Arlington Heights, IL, USA), respectively. Body composition was assessed using dual‐energy X‐ray absorptiometry (GE Lunar Prodigy, GE Healthcare Technologies Inc., Chicago, IL, USA). Basal metabolic rate was measured by indirect calorimetry (VIASYS Healthcare Inc., Conshohocken, PA, USA) following a 12‐h overnight fast in a thermoneutral, darkened environment, with participants resting supine for 30 min. Daily energy expenditure was estimated by multiplying the measured basal metabolic rate by a physical activity factor of 1.375 (Harris & Benedict, 1918). Participants were also exposed to intermittent hypoxaemia (≤ 20 min) using the experimental apparatus described below and monitored for symptoms of acute mountain sickness using the Lake Louise scoring system (e.g., nausea, fatigue, headache). All participants tolerated the exposure without adverse events.

### 2.5 Experimental sessions: Normoxaemia and intermittent hypoxaemia

Upon arrival for each experimental session, resting blood pressure was measured using an automated sphygmomanometer (American Diagnostic Corporation, Hauppauge, NY, USA). An intravenous catheter was inserted into an antecubital vein, and a baseline blood sample was collected. Participants then consumed a high‐fat meal providing 33% of estimated daily energy expenditure (59% fat, 31% carbohydrate, 10% protein), composed of Ensure Plus (Abbott Laboratories, Lake County, IL, USA) and 35% whipping cream (Sealtest, Longueil, Canada). Water was available ad libitum throughout the session. Participants remained awake in a semi‐recumbent position for 6 h and were continuously monitored using a fingertip pulse oximeter (Masimo, Irvine, CA, USA), with heart rate and peripheral oxygen saturation ($S_{pO_{2}}$) recorded at 1‐s intervals.

Following meal consumption, participants were exposed for 6 h to normoxaemia (ambient air: fraction of inspired oxygen [$F_{iO_{2}}$] ∼20.93%, $S_{pO_{2}}$ ∼98%) or intermittent hypoxaemia, consisting of 15 hypoxaemic cycles/h initiated at 4‐min intervals, achieved by inhalation of 100% medical‐grade nitrogen (Messer, Missisauga, Canada) via an oronasal mask with a two‐way non‐rebreathing valve. Nitrogen inhalation continued until $S_{pO_{2}}$ reached 85%, after which the inspiratory line was switched to ambient air, where $S_{pO_{2}}$ returned to baseline typically within approximately 10–40 s. This frequency approximates a moderate apnoea–hypopnea index (15 events/h), a common metric of obstructive sleep apnoea severity. Inspiratory line switching was performed manually based on continuous visual monitoring of $S_{pO_{2}}$. The order of experimental conditions was randomized, with four participants completing the intermittent hypoxaemia condition first. Mild symptoms of acute mountain sickness were reported by one participant following intermittent hypoxaemia and by one participant following normoxaemia.

### 2.6 Follow‐up session: Continuous hypoxaemia

**FIGURE A1:**
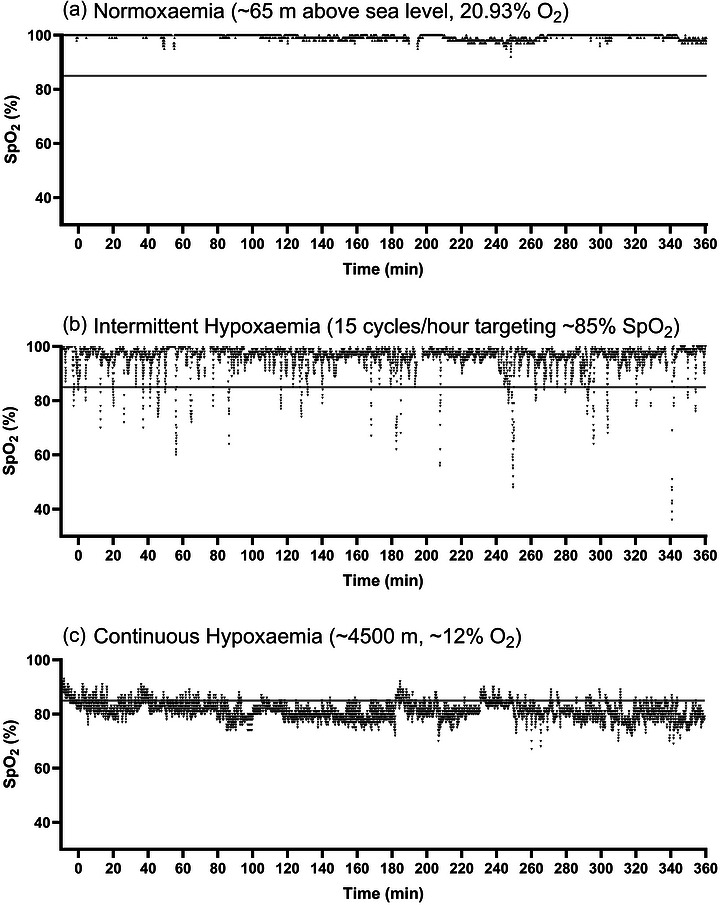
Peripheral oxygen saturation ($S_{pO_{2}}$) measured over 6 h in a healthy young female adult participant during normoxaemia (65 m above sea level; 20.93% fraction of inspired oxygen; panel a), intermittent hypoxaemia (15 hypoxaemic cycles per hour, initiated at 4‐min intervals, achieved by inhalation of 100% medical‐grade nitrogen and targeting 85% $S_{pO_{2}}$; panel b) and continuous hypoxaemia (programmed oxygen extractors to simulate an altitude of ∼5000 m; achieved fraction of inspired oxygen of ∼12%, corresponding to simulated altitude of ∼4500 m; panel c) in a climate‐controlled environmental chamber regulated at 20–22°C and 30% relative humidity. The continuous grey line represents an $S_{pO_{2}}$ of 85%. Data were recorded in 1‐s intervals using a fingertip pulse oximeter

After completing the primary trial, participants were invited to complete an additional experimental session involving continuous hypoxaemia. Eight participants consented to this follow‐up; four declined due to scheduling constraints. All pre‐experimental procedures were identical to the primary protocol, except that sessions were not restricted to a specific menstrual cycle phase. During this condition, participants were exposed for 6 h to continuous normobaric hypoxia in a climate‐controlled hypoxic chamber with oxygen extractors (Altitude Control Technologies, Edwards, CO, USA), regulated at 20–22°C and 30% relative humidity. The hypoxic chamber was programmed to simulate an altitude of ∼5000 m. The mean $F_{iO_{2}}$ achieved over the 6‐h exposure was ∼12.0%, corresponding to a simulated altitude of approximately 4500 m. The deviation from the programmed altitude reflects the inflow of ambient air associated with periodic researcher entry and exit during the protocol (e.g., for blood sample collection), which the chamber's oxygen extractors cannot fully compensate for in real time. A representative individual $S_{pO_{2}}$ response across all three experimental conditions is included in Figure A1 for visualization of the hypoxic stimulus. Two participants reported mild symptoms consistent with acute mountain sickness during this continuous hypoxaemia exposure.

### 2.7 Blood sample collection

Venous blood samples were collected at baseline and 30, 60, 90, 120, 180, 240, 300 and 360 min post‐meal into EDTA‐treated and serum tubes (BD Vacutainer, Franklin Lakes, NJ, USA). Plasma and serum were separated by centrifugation at 1250 RCF for 10 min at 4°C. Serum samples were allowed to clot for ∼30 min at room temperature before centrifugation. Aliquots were stored at −80°C until analysis.

### 2.8 Analyte assays

Serum concentrations of progesterone and β‐oestradiol were measured at baseline, whereas plasma concentrations of glucose, NEFA, triglycerides and insulin were measured at baseline and 30, 60, 90, 120, 180, 240, 300 and 360 min post‐meal. Plasma BHB concentrations were measured hourly (baseline and 60–360 min post‐meal) and were not assessed at the 30‐ or 90‐min time points. All samples were quantified in duplicate using commercially available colorimetric assays, following the manufacturer's protocols, as previously described by our group (Goulet, Marcoux et al., 2024; Marcoux et al., 2022).

### 2.9 Sample size determination

The parent trial was powered to detect differences in plasma triglyceride concentrations. An a priori power analysis was performed using G*Power (v.3.1.9.7) and a moderate effect size (*F* = 0.250), with a correlation among repeated measures of 0.5, a non‐sphericity correction of 1.0, a total of nine measurements, and two groups. The power analysis revealed that a total sample size of 22 participants was required to achieve a power of 0.95 with α set at 0.050 to identify a significant within–between interaction. The parent trial had a sample size of 24 participants (12 females, 12 males). Data for BHB responses in male participants are published elsewhere (Marcoux et al., 2022).

### 2.10 Statistical analysis

Physiological variables (blood pressure, heart rate and $S_{pO_{2}}$), serum concentrations of progesterone and β‐oestradiol and baseline plasma concentrations of BHB and other metabolites (glucose, NEFA and triglycerides) and insulin were analysed using linear mixed‐effects models with condition (normoxaemia, intermittent hypoxaemia and continuous hypoxaemia) as a fixed effect and participant identification as a random intercept. Postprandial concentrations of plasma BHB, other metabolites and insulin were analysed using linear mixed‐effects models with condition and time (30, 60, 90, 120, 180, 240, 300 and 360 min post‐meal) as fixed effects, and participant identification as a random intercept, while baseline (0 min) was included as a continuous covariate. Differences in BHB concentrations between baseline and 360 min post‐meal were analysed using a linear mixed‐effects model with time and condition as fixed effects and participant identification as a random intercept.

Descriptive data for each condition are presented as means and standard deviations of raw data. Within‐ and between‐condition differences are presented as model‐derived estimated marginal means with 95% confidence intervals (CI). Bonferroni's post hoc comparisons were performed when a significant main effect or interaction was identified. Homoskedasticity and normality of the residuals were assessed using diagnostic plots. Statistical analyses were performed in *jamovi* (v2.5.6; *gamlj3* module v3.3.4), with an α‐level of 0.050. Figures were generated using GraphPad Prism (v10.6.1, GraphPad Software, Boston, USA).

## 3 RESULTS

Participant characteristics at enrolment for the primary group and the follow‐up subgroup are presented in Table 1. Baseline serum concentrations of progesterone and β‐oestradiol are presented in Table 2 and were similar across experimental conditions (both *P* ≥ 0.155).

**TABLE 1:** Characteristics of the primary investigation group and the follow‐up session subgroup.

Characteristic	Primary investigation group (*n* = 12)	Follow‐up session subgroup (*n* = 8)
Age (years)	21.3 (3.17)	21.0 (3.21)
Height (m)	1.66 (0.07)	1.65 (0.07)
Weight (kg)	62.9 (12.8)	62.0 (15.4)
Waist (cm)	71.8 (8.56)	71.3 (10.4)
BMI (kg/m^2^)	22.7 (3.54)	22.7 (4.23)
Lean mass (kg)	41.0 (7.37)	39.9 (8.21)
Fat mass (kg)	18.8 (5.97)	18.7 (7.05)
Fat mass (%)	31.1 (5.96)	31.4 (6.59)
BMR (kcal)	1455 (317)	1465 (368)
PSQIS	7.8 (4.4)	7.0 (5.0)

*Note*: Data are presented as means (standard deviations) of raw data. The primary investigation group completed the experimental protocols with exposures to normoxaemia and intermittent hypoxaemia. A subset of participants completed an additional follow‐up session with exposure to continuous hypoxaemia. Abbreviations: BMI, body mass index; BMR, basal metabolic rate; PSQIS, Pittsburgh Sleep Questionnaire Index Score.

**TABLE 2:** Serum concentrations of progesterone and β‐oestradiol per experimental condition

Steroid sex hormone	Normoxaemia	Intermittent hypoxaemia	Continuous hypoxaemia	*P*
Progesterone (ng/mL)	1.65 (1.6)	2.76 (4.2)	5.00 (5.2)	0.155
Oestradiol (pg/mL)	90.7 (47)	92.2 (42)	73.5 (47)	0.244

*Note*: Data are presented as means (standard deviations) of raw data and were compared using linear mixed‐effects models with condition as a fixed effect and participant identification as a random intercept with an α‐level of 0.050. The analyses of serum β‐oestradiol concentrations exclude one outlier (normoxaemia: 652 pg/mL; intermittent hypoxaemia: 822 pg/mL), who had values >14 standard deviations above the group mean. The same participant also displayed serum progesterone concentrations above the 95th percentile (normoxaemia, 5.41 ng/mL; intermittent hypoxaemia, 6.00 ng/mL) (Stricker et al., 2006).

### 3.1 Physiological responses to 6 h of exposure to normoxaemia, intermittent hypoxaemia and continuous hypoxaemia

Table 3 presents the mean systolic and diastolic blood pressures, heart rate and $S_{pO_{2}}$ during normoxaemia, intermittent hypoxaemia and continuous hypoxaemia. Systolic and diastolic blood pressures were similar between normoxaemia, intermittent hypoxaemia and continuous hypoxaemia (both *P* ≥ 0.201). Heart rate differed across conditions (*P* < 0.001), with higher values observed during continuous hypoxaemia (95.0 bpm; 95% CI: 87.5–102.5) compared with normoxaemia (76.9 bpm; 95% CI: 70.6–83.2; *P*_Bonferroni_ < 0.001) and intermittent hypoxaemia (79.8 bpm; 95% CI: 73.5–86.1; *P*_Bonferroni_ < 0.001), whereas normoxaemia and intermittent hypoxaemia did not differ (*P*_Bonferroni_ = 0.631). Mean $S_{pO_{2}}$ also differed across experimental conditions (*P* < 0.001), being lowest during continuous hypoxaemia (83.9%; 95% CI: 82.2–85.6) compared with normoxaemia (97.8%; 95% CI: 96.7–98.9; *P*_Bonferroni_ < 0.001) and intermittent hypoxaemia (96.1%; 95% CI: 94.9–97.2; *P*_Bonferroni_ < 0.001), which did not differ from each other (*P*_Bonferroni_ = 0.102). Time spent below $S_{pO_{2}}$ thresholds (≤90%, ≤85% and ≤80%) increased stepwise from normoxaemia to intermittent hypoxaemia to continuous hypoxaemia (all *P* < 0.001).

**TABLE 3:** Mean systolic and diastolic blood pressures, heart rate and peripheral oxygen saturation following the consumption of a high‐fat meal during 6 h of exposure to normoxaemia, intermittent hypoxaemia and continuous hypoxaemia in young adult females

Physiological variable	Normoxaemia	Intermittent hypoxaemia	Continuous hypoxaemia	*P*
SBP (mmHg)	112 (9.1)	112 (8.9)	107 (7.4)	0.499
DBP (mmHg)	60.2 (4.0)	61.4 (3.9)	58.0 (2.0)	0.201
Heart rate (bpm)	76.8 (9.1)	80.1 (10.4)	93.2 (13.2)†‡	<0.001
$S_{pO_{2}}$ (%)	97.8 (1.3)	96.1 (2.2)	83.9 (2.2)†‡	<0.001
Time $S_{pO_{2}}$ ≤ 90% (min)	4.16 (6.3)	30.6 (15.6)*	324 (45.7)†‡	<0.001
Time $S_{pO_{2}}$ ≤ 85% (min)	1.81 (2.6)	17.7 (10.6)	245 (62.7)†‡	<0.001
Time $S_{pO_{2}}$ ≤ 80% (min)	0.55 (0.89)	10.3 (7.4)	71.9 (56.9)†‡	<0.001

*Note*: Data are presented as means (standard deviations) of raw data and were compared using linear mixed‐effects models with condition as a fixed effect and participant identification as a random intercept with an α‐level of 0.050. Pairwise comparisons were performed using Bonferroni's post hoc test. ^*^Statistically significant difference between normoxaemia and intermittent hypoxaemia. ^†^Statistically significant difference between normoxaemia and continuous hypoxaemia. ^‡^Statistically significant difference between intermittent hypoxaemia and continuous hypoxaemia. Abbreviations: DBP, diastolic blood pressure; SBP, systolic blood pressure; $S_{pO_{2}}$, peripheral oxygen saturation.

### 3.2 Plasma BHB response to a high‐fat meal during 6 h of exposure to normoxaemia, intermittent hypoxaemia and continuous hypoxaemia

**FIGURE 1:**
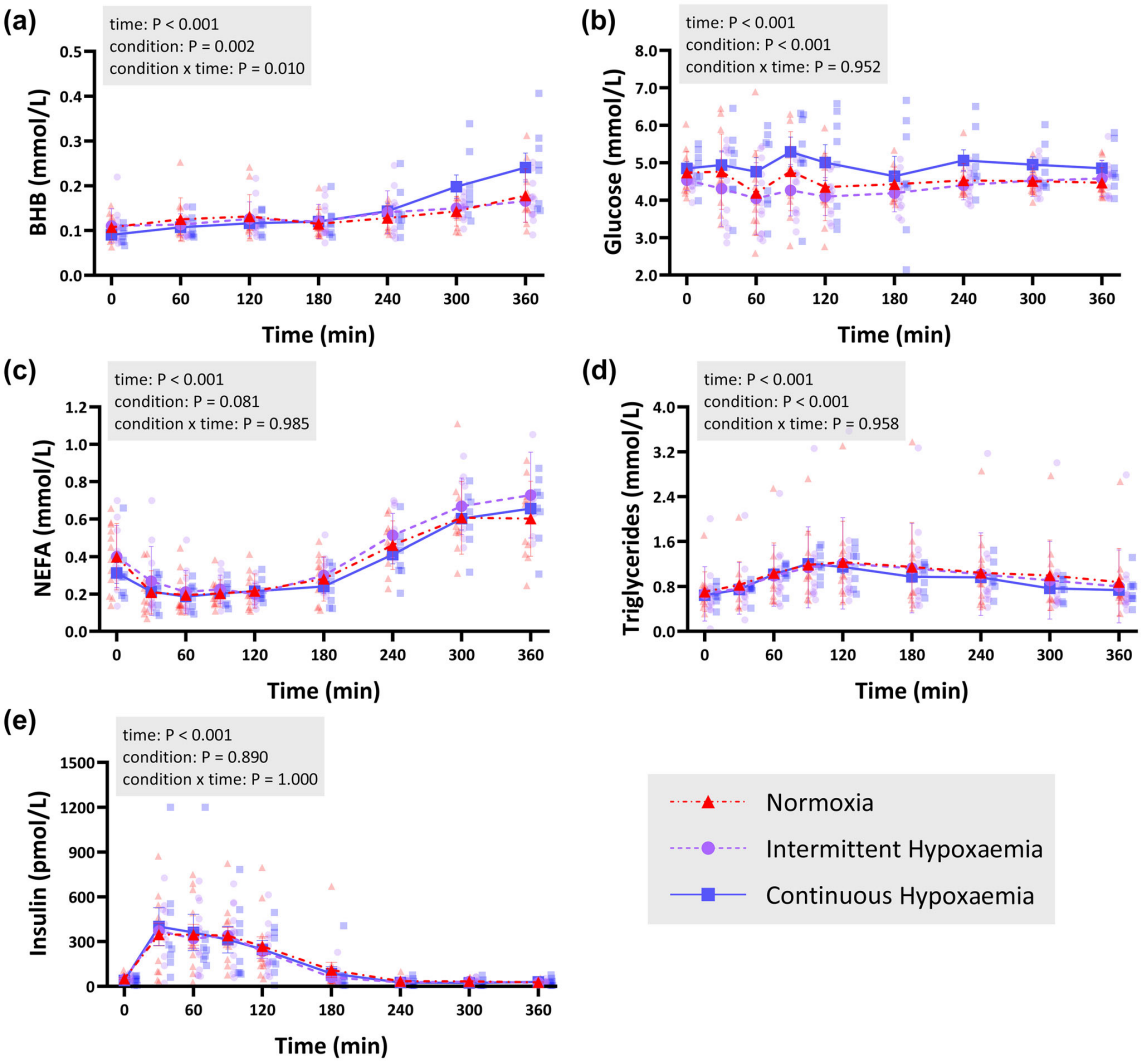
Plasma concentrations of BHB (a), glucose (b), NEFA (c), triglycerides (d) and insulin (e) in young adult females at baseline (0 min) and every hour after consuming a high‐fat meal during 6 h of exposure to normoxaemia (red), intermittent hypoxaemia (purple) and continuous hypoxaemia (blue). Data are presented as means (standard deviations) of raw data and individual points. Postprandial data were analysed using linear mixed‐effects models with condition and time as fixed effects, participant identification as a random intercept and baseline as a continuous covariate. Note: Model‐derived estimated marginal means with 95% confidence intervals are presented in Figure A2 for visualization of baseline‐adjusted condition‐level effects. BHB, β‐hydroxybutyrate; NEFA, non‐esterified fatty acid.

Figure 1a presents baseline and postprandial plasma BHB concentrations. Baseline plasma BHB concentrations were similar across experimental conditions (normoxaemia: 0.107 mmol/L; 95% CI: 0.089–0.125; intermittent hypoxaemia: 0.110 mmol/L; 95% CI: 0.092–0.128; continuous hypoxaemia: 0.089; 95% CI: 0.068–0.110; *P* = 0.167). A time × condition interaction was observed for postprandial BHB concentrations (*P* = 0.010), indicating that the temporal pattern of the BHB response differed across conditions. Within‐condition comparisons showed that BHB concentrations increased over the postprandial period only during continuous hypoxaemia, reaching 80.5% and 119% higher concentrations at 300 min (0.204 mmol/L; 95% CI: 0.175–0.232; *P*_Bonferroni_ = 0.002) and 360 min (0.247 mmol/L; 95% CI: 0.218–0.275; *P*_Bonferroni_ < 0.001), respectively, than at 60 min (0.113 mmol/L; 95% CI: 0.084–0.142). No within‐condition differences were observed at any other time points across all conditions (all *P*_Bonferroni_ ≥ 0.234). Between‐condition comparisons at each time point indicated that BHB concentrations at 360 min were 13.0% and 14.2% higher during continuous hypoxaemia compared with the corresponding time point under normoxaemia (0.176 mmol/L; 95% CI: 0.153–0.200; *P*_Bonferroni_ = 0.029) and intermittent hypoxaemia (0.163 mmol/L; 95% CI: 0.139–0.186; *P*_Bonferroni_ = 0.002), respectively. No between‐condition differences were observed at earlier postprandial time points (all *P*_Bonferroni_ ≥ 0.135).

A time × condition interaction (*P* = 0.003) was also observed when comparing BHB concentrations at baseline and 360 min post‐meal. Post hoc analyses showed that BHB concentrations were 66.4%, 50.9% and 172% higher after 360 min than at baseline during normoxaemia (0 min vs. 360 min: 0.107 mmol/L; 95% CI: 0.078–0.137; vs. 0.178 mmol/L; 95% CI: 0.149–0.207; *P*_Bonferroni_ = 0.003), intermittent hypoxaemia (0.110 mmol/L; 95% CI: 0.081–0.140; vs. 0.166 mmol/L; 95% CI: 0.136–0.195; *P*_Bonferroni_ = 0.042) and continuous hypoxaemia (0.088 mmol/L; 95% CI: 0.053–0.123; vs. 0.239 mmol/L; 95% CI: 0.204–0.273; *P*_Bonferroni_ < 0.001), respectively.

### 3.3 Plasma metabolic and insulinemic responses to a high‐fat meal during 6 h of exposure to normoxaemia, intermittent hypoxaemia and continuous hypoxaemia

Figure 1b–e presents baseline and postprandial plasma concentrations of glucose, NEFA, triglycerides and insulin. Baseline plasma metabolite and insulin concentrations were similar across experimental conditions (all *P* ≥ 0.088). More precisely, baseline plasma glucose concentrations averaged 4.74 mmol/L during normoxaemia (95% CI: 4.46–5.02), 4.53 mmol/L during intermittent hypoxaemia (95% CI: 4.26–4.81) and 4.92 mmol/L during continuous hypoxaemia (95% CI: 4.60–5.24; *P* = 0.088). Baseline plasma NEFA concentrations averaged 0.399 mmol/L during normoxaemia (95% CI: 0.298–0.499), 0.381 mmol/L during intermittent hypoxaemia (95% CI: 0.280–0.481) and 0.317 mmol/L during continuous hypoxaemia (95% CI: 0.197–0.438; *P* = 0.492). Baseline plasma triglyceride concentrations averaged 0.764 mmol/L during normoxaemia (95% CI: 0.522–1.005), 0.741 mmol/L during intermittent hypoxaemia (95% CI: 0.499–0.983) and 0.553 mmol/L during continuous hypoxaemia (95% CI: 0.288–0.818; *P* = 0.164). Baseline plasma insulin concentrations averaged 51.2 pmol/L during normoxaemia (95% CI: 37.4–64.9), 48.2 pmol/L during intermittent hypoxaemia (95% CI: 34.5–61.9) and 41.4 pmol/L during continuous hypoxaemia (95% CI: 25.8–57.0; *P* = 0.444).

For plasma glucose concentrations (Figure 1b), no main effect of time (*P* = 0.231) or time × condition interaction (*P* = 0.952) was observed. A main effect of condition was detected (*P* < 0.001), and post hoc comparisons revealed that mean postprandial glucose concentrations were higher by 9.8% and 12.5% during continuous hypoxaemia (4.92 mmol/L; 95% CI: 4.65–5.18) compared to normoxaemia (4.48 mmol/L; 95% CI: 4.25–4.71; *P*_Bonferroni_ = 0.001) and intermittent hypoxaemia (4.37 mmol/L; 95% CI: 4.14–4.60; *P*_Bonferroni_ < 0.001), respectively, whereas normoxaemia and intermittent hypoxaemia were similar (*P*_Bonferroni_ = 0.945). For plasma NEFA concentrations (Figure 1c), a main effect of time was observed (*P* < 0.001), characterized by an early postprandial suppression followed by a progressive rebound later in the postprandial period. There was no main effect of condition (*P* = 0.081) nor time × condition interaction (*P* = 0.985), indicating similar NEFA responses across experimental conditions. More precisely, postprandial plasma NEFA concentrations averaged 0.338 mmol/L during normoxaemia (95% CI: 0.308–0.368), 0.377 mmol/L during intermittent hypoxaemia (95% CI: 0.347–0.407) and 0.353 mmol/L during continuous hypoxaemia (95% CI: 0.317–0.389).

For plasma triglyceride concentrations (Figure 1d), a main effect of time was observed (*P* < 0.001), reflecting the postprandial rise and subsequent decline following the high‐fat meal. A main effect of condition was also detected (*P* < 0.001); however, there was no time × condition interaction (*P* = 0.958). Post hoc pairwise comparisons showed that, when averaged across the postprandial period, a 14.1% and 13.9% increase in triglyceride concentrations was observed during continuous hypoxaemia (1.095 mmol/L; 95% CI: 0.996–1.190) compared to normoxaemia (0.960 mmol/L; 95% CI: 0.870–1.050; *P*_Bonferroni_ = 0.002) and intermittent hypoxaemia (0.961 mmol/L; 95% CI: 0.871–1.050; *P*_Bonferroni_ = 0.001), respectively, with no difference between normoxaemia and intermittent hypoxaemia (*P*_Bonferroni_ = 1.000). For plasma insulin concentrations (Figure 1e), a main effect of time (*P* < 0.001) was observed, whereas no main effect of condition (*P* = 0.890) or time × condition interaction (*P* = 1.000) was observed. More precisely, postprandial plasma insulin concentrations averaged 185 pmol/L during normoxaemia (95% CI: 125–246), 176 pmol/L during intermittent hypoxaemia (95% CI: 115–236) and 183 pmol/L during continuous hypoxaemia (95% CI: 119–248).

## 4 DISCUSSION

This study evaluated the effects of normoxaemia, intermittent hypoxaemia and continuous hypoxaemia on postprandial plasma BHB concentrations and related cardiometabolic markers in young adult females following a high‐fat meal. The primary and novel finding is that continuous hypoxaemia, but not intermittent hypoxaemia, resulted in higher postprandial BHB concentrations than normoxaemia, particularly during the late postprandial period (6 h post‐meal). This elevation occurred alongside higher postprandial glucose concentrations and marginally, but statistically, higher postprandial triglyceride concentrations, whereas NEFA and insulin concentrations remained similar across experimental conditions. Collectively, these findings suggest that sustained reductions in oxygen availability acutely alter postprandial metabolism in young adult females.

### 4.1 The effect of normoxaemia, intermittent hypoxaemia and continuous hypoxaemia on postprandial plasma BHB concentrations after a high‐fat meal

Our observation that a high‐fat meal increases plasma BHB concentrations above baseline under normoxaemia is consistent with previous reports. For example, Halkes et al. (2003) and Meijssen et al. (2000) demonstrated that an oral fat‐loading test increased BHB concentrations, with greater increases observed in individuals with familial combined hyperlipidaemia, a disorder characterized by impaired postprandial triglyceride and NEFA clearance. These findings suggest that greater hepatic NEFA delivery during the postprandial period promotes hepatic ketone body production. Notably, these increases occur despite the concomitant rise in insulin and suppression of circulating NEFA, which may inhibit ketogenesis (Cotter et al., 2013). Whether postprandial changes in BHB clearance also contribute to this response remains unclear.

The present study extends these observations by demonstrating that 6 h of continuous hypoxaemia increases late postprandial plasma BHB concentrations in young adult females after a high‐fat meal, compared with normoxaemia and intermittent hypoxaemia, despite similar circulating NEFA and insulin responses across conditions. This design allowed a direct comparison of hypoxaemia patterns under matched postprandial conditions, which was not possible in our previous work (Marcoux et al., 2022). In that study, continuous hypoxaemia increased BHB concentrations in young adult males during a 6‐h fast, whereas intermittent hypoxaemia in the postprandial state had no statistically significant effect compared with normoxaemia (Marcoux et al., 2022). However, differences in nutritional status precluded direct comparisons of hypoxaemia patterns, and the statistical approach used a repeated‐measures ANOVA rather than a linear mixed‐effects model. The concomitant elevations in postprandial plasma triglyceride and glucose concentrations during continuous hypoxaemia may help explain the BHB response observed in the present study. Elevated postprandial triglyceride concentrations may reflect impaired systemic triglyceride clearance or increased triglyceride production, both of which may indicate greater hepatic NEFA availability, which could lead to increased hepatic BHB production (Cotter et al., 2013). In parallel, elevated postprandial glucose concentrations may reflect subtle alterations in hepatic glucose uptake, storage, or production, or in insulin sensitivity, that, although insufficient to alter circulating insulin concentrations, could influence hepatic substrate partitioning (Newhouse et al., 2017; Woolcott et al., 2015). Together, these findings suggest that continuous hypoxaemia alters postprandial BHB concentrations through mechanisms not fully explained by circulating NEFA or insulin concentrations alone.

In contrast to continuous hypoxaemia, exposure to 6 h of intermittent hypoxaemia did not result in higher postprandial plasma BHB concentrations relative to normoxaemia. The lack of effect under intermittent hypoxaemia may be partly explained by inherent differences in hypoxaemic dose (i.e., different levels of oxygen deprivation) or by the distinct molecular mechanisms, such as the regulation of hypoxia‐inducible factor, that are unique to intermittent hypoxaemia as opposed to those regulated by continuous hypoxaemia (Hunyor & Cook, 2018). However, these explanations remain speculative and further research is warranted. Additionally, sex‐specific metabolic responses may further modulate postprandial BHB responses. For example, previous findings show that females have higher postprandial BHB concentrations than males, potentially due to greater postprandial hepatic NEFA oxidation in females (Halkes et al., 2003).

### 4.2 Perspective

The present findings are particularly relevant as an increase in circulating BHB during sustained hypoxaemia may be metabolically advantageous. Hypoxic exposure is well recognized to elevate resting metabolic rate and to contribute to the unintentional weight loss frequently observed in mountaineers ascending to high altitude (Kayser & Verges, 2013). In this context of increased energy demand and reduced oxygen availability, BHB serves as an efficient oxidative substrate, yielding more ATP per molecule of oxygen consumed than either glucose or fatty acids in vivo (Veech, 2004). A shift toward greater ketone body utilization could therefore help preserve cellular energy production when oxygen supply is constrained. While the present findings cannot directly address ketone utilization, the elevated postprandial BHB concentrations observed during continuous hypoxaemia are consistent with this framework and warrant further investigation into whether hypoxaemia‐induced increases in circulating BHB confer functional metabolic benefits in humans.

### 4.3 Limitations and future research directions

Several limitations and future research directions should be acknowledged. First, this study represents a secondary analysis of a larger randomized crossover trial, and an a priori power calculation was not performed for BHB responses. As such, these findings should be interpreted as exploratory and hypothesis‐generating. Despite this, several between‐condition differences were statistically significant, and the corresponding percentage changes and 95% confidence intervals may help inform sample size estimates for future confirmatory studies. Second, the continuous hypoxaemia condition was conducted as part of a follow‐up session and was therefore not randomized nor restricted to a single menstrual cycle phase; however, serum oestradiol and progesterone concentrations did not differ between conditions. It remains unclear whether menstrual cycle phases influence postprandial BHB responses. Third, participants were not blinded to the experimental conditions and may have experienced altered stress or arousal, which could have confounded the autonomic nervous system response to hypoxaemia. Finally, an additional consideration is that the intermittent and continuous hypoxaemia protocols differed not only in the temporal pattern of exposure but also in the depth of desaturation. Although the difference in the depth was modest (6‐h mean $S_{pO_{2}}$ of 83.9% during continuous hypoxaemia versus a cycle target of ∼85% during intermittent hypoxaemia; Table 3), participants under the intermittent protocol spent the majority of the 6‐h exposure in normoxaemia between cycles, whereas participants under the continuous protocol were exposed to sustained hypoxaemia throughout. The overall hypoxaemic dose was therefore substantially greater under the continuous protocol, driven mainly by the duration of desaturation rather than its depth. Future studies employing dose‐matched protocols will be required to disentangle the relative contributions of hypoxaemic pattern and hypoxaemic severity to postprandial BHB responses. Whether these laboratory‐based observations translate to ecologically relevant settings, such as active high‐altitude ascent or sleep‐related hypoxaemia in free‐living individuals with obstructive sleep apnoea, also remains to be determined.

### 4.4 Conclusion

In conclusion, this study demonstrates that continuous hypoxaemia, but not intermittent hypoxaemia, increases postprandial plasma BHB concentrations in young adult females following a high‐fat meal, relative to normoxaemia. This effect emerged primarily during the late postprandial period and occurred independently of changes in plasma NEFA and insulin concentrations, two key regulators of BHB production. More broadly, these findings highlight the importance of considering the characteristics of hypoxaemic exposure, including its pattern and dose, when evaluating postprandial metabolic responses under hypoxaemia. Future studies are warranted to investigate how hypoxaemia may alter circulating ketone bodies and to determine whether elevated BHB concentrations confer metabolic advantages when oxygen supply is limited in humans.

## AUTHOR CONTRIBUTIONS

Nicholas Goulet: Methodology; formal analysis; investigation; writing – original draft; writing – review and editing; visualization. Alexanne Laroque: Formal analysis; investigation; writing – original draft; writing – review and editing. Caroline Marcoux: Investigation; writing – review and editing. Vincent Bourgon: Investigation; writing – review and editing. Jean‐François Mauger: Methodology; investigation; writing – review and editing. Ruwan Amaratunga: Funding acquisition; writing – review and editing. Pascal Imbeault: Conceptualization; methodology; writing – review and editing. All authors approved the final version of the manuscript; agree to be accountable for all aspects of the work in ensuring that questions related to the accuracy or integrity of any part of the work are appropriately investigated and resolved; and all persons designated as authors qualify for authorship, and all those who qualify for authorship are listed.

## CONFLICT OF INTEREST

None declared.

## GENERATIVE AI STATEMENT

During the preparation of this manuscript, the authors used Claude (Anthropic; Claude Opus 4.5 and Claude Opus 4.7) and Grammarly (Grammarly Inc.) to support the editorial refinement of human‐authored text after the authors' initial drafting. Specifically, these tools were used to refine the wording of selected passages, suggest structural revisions, check grammar and style, and assist with French‐to‐English translation. These tools were not used to generate scientific content, conduct or interpret data analyses, or produce any of the underlying study data. All AI‐assisted text was reviewed, edited and approved by the authors, who take full responsibility for the content of the manuscript.

## Figures and Tables

**FIGURE A2:**
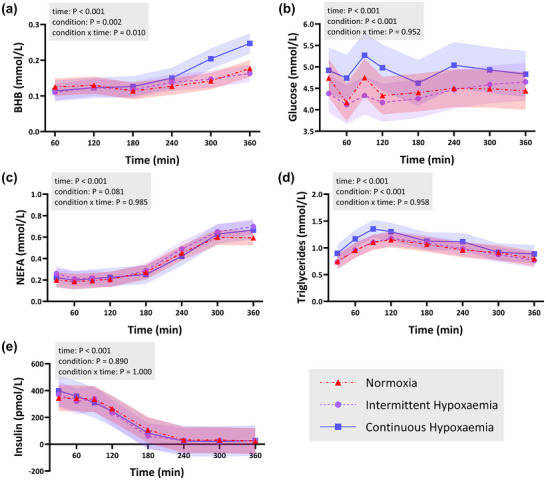
Postprandial plasma concentrations of BHB (a), glucose (b), NEFA (c), triglycerides (d), and insulin (e) in young adult females after consuming a high‐fat meal during 6 h of exposure to normoxaemia (red), intermittent hypoxaemia (purple) and continuous hypoxaemia (blue). Data are presented as model‐derived estimated marginal means (95% confidence intervals). Postprandial data were analysed using linear mixed‐effects models with condition and time as fixed effects, participant identification as a random intercept and baseline (0 min) as a continuous covariate. BHB, β‐hydroxybutyrate; NEFA, non‐esterified fatty acid.

## Data Availability

The datasets generated for this study are available upon reasonable request to the corresponding author and with a signed access agreement.
